# Integration of point-of-care screening for type 2 diabetes mellitus and hypertension into the COVID-19 vaccine programme in Johannesburg, South Africa

**DOI:** 10.1186/s12889-023-17190-6

**Published:** 2023-11-20

**Authors:** Alana T Brennan, Beatrice Vetter, Sithabiso D Masuku, Bukelwa Mtshazo, Nkuli Mashabane, Simiso Sokhela, Willem DF Venter, Kekeletso Kao, Gesine Meyer-Rath

**Affiliations:** 1 Department of Global Health, Boston University School of Public Health, Boston, MA USA; 2 Health Economics and Epidemiology Research Office, Faculty of Health Sciences, University of the Witwatersrand, Johannesburg, South Africa; 3 Department of Epidemiology, Boston University School of Public Health, Boston, MA USA; 4 FIND, Geneva, Switzerland; 5 Wits Ezintsha, Faculty of Health Sciences, University of the Witwatersrand, Johannesburg, South Africa

**Keywords:** Opportunistic screening, COVID-19 vaccine, Hypertension, Diabetes, Obesity, Metabolic syndrome, South Africa

## Abstract

**Background:**

South Africa grapples with a substantial burden of non-communicable diseases (NCDs), particularly type 2 diabetes (diabetes) and hypertension. However, these conditions are often underdiagnosed and poorly managed, further exacerbated by the strained primary healthcare (PHC) system and the disruptive impact of the COVID-19 pandemic. Integrating NCD screening with large-scale healthcare initiatives, such as COVID-19 vaccination campaigns, offers a potential solution, especially in low- and middle-income countries (LMICs). We investigated the feasibility and effectiveness of this integration.

**Methods:**

A prospective cohort study was conducted at four government health facilities in Johannesburg, South Africa. NCD screening was incorporated into the COVID-19 vaccination campaign. Participants underwent COVID-19 rapid tests, blood glucose checks, blood pressure assessments, and anthropometric measurements. Those with elevated blood glucose or blood pressure values received referrals for diagnostic confirmation at local PHC centers.

**Results:**

Among 1,376 participants screened, the overall diabetes prevalence was 4.1%, combining previously diagnosed cases and newly identified elevated blood glucose levels. Similarly, the hypertension prevalence was 19.4%, comprising pre-existing diagnoses and newly detected elevated blood pressure cases. Notably, 46.1% of participants displayed waist circumferences indicative of metabolic syndrome, more prevalent among females. Impressively, 7.8% of all participants screened were potentially newly diagnosed with diabetes or hypertension. Approximately 50% of individuals with elevated blood glucose or blood pressure successfully linked to follow-up care within four weeks.

**Conclusion:**

Our study underscores the value of utilizing even brief healthcare interactions as opportunities for screening additional health conditions, thereby aiding the identification of previously undiagnosed cases. Integrating NCD screenings into routine healthcare visits holds promise, especially in resource-constrained settings. Nonetheless, concerted efforts to strengthen care linkage are crucial for holistic NCD management and control. These findings provide actionable insights for addressing the NCD challenge and improving healthcare delivery in LMICs.

**Supplementary Information:**

The online version contains supplementary material available at 10.1186/s12889-023-17190-6.

## Introduction

The burden of non-communicable diseases (NCDs) in South Africa has reached alarming levels, accounting for 51% of all deaths in the country [1]. Among the various NCDs, type 2 diabetes mellitus (diabetes) and hypertension stand out as major health concerns. Diabetes is associated with multiple risk factors and complications, including hypertension, dyslipidemia, cardiovascular diseases, and chronic kidney disease. Unfortunately, both diabetes and its related conditions are often underdiagnosed and poorly managed in South Africa. Recent prevalence studies have revealed a diabetes prevalence of 10.1% and a hypertension prevalence of 35.1% in the country, indicating a significant public health challenge [2–4]. Additionally, a considerable proportion of the population is found to have pre-diabetic conditions, further emphasizing the urgent need for improved care [5]. Despite existing screening guidelines, many individuals with diabetes and hypertension remain undetected, undiagnosed, or inadequately treated, highlighting the critical gap in healthcare provision [3].

The primary healthcare (PHC) system in South Africa faces formidable challenges in providing comprehensive screening and care for NCDs due to the overwhelming workload it carries. Consequently, a significant number of individuals with elevated blood glucose and/or blood pressure levels remain unaware of their condition or lack proper management [6]. While community-based routine screening has proven effective for other health conditions like tuberculosis and HIV in sub-Saharan Africa, comparable initiatives targeting NCDs are lacking [7, 8]. The recent COVID-19 pandemic has further exacerbated healthcare seeking and screening for NCDs, disrupting services and restricting public access to essential care [9]. To bridge this gap, there was a suggestion to leverage existing COVID-19 control efforts, such as vaccination campaigns, as an opportunity to integrate screening for NCDs. However, the feasibility and effectiveness of such integrated screening programs, particularly in low- and middle-income countries (LMICs), remain inadequately studied [10]. Moreover, it is unclear to what extent individuals identified as having an elevated risk for NCDs would actively seek further care and treatment for their condition.

In a previous study we conducted [11], we successfully demonstrated the feasibility of utilizing the existing COVID-19 screening infrastructure in South Africa to conduct opportunistic screening for diabetes and hypertension. During our previous study, we screened a total of 1169 participants and identified a significant proportion of previously undiagnosed cases. Approximately 22% of the participants received a potential new diagnosis of diabetes and/or hypertension through this opportunistic screening. Building upon these promising results, our current study aimed to assess the feasibility, effectiveness, and linkage-to-care of implementing a similar rapid screening program for diabetes, hypertension, and COVID-19 in conjunction with an ongoing government COVID-19 vaccination program. Our approach involved leveraging the existing COVID-19 vaccine infrastructure at public sector vaccination sites, thus creating efficient opportunities for the rapid screening of diabetes, hypertension and COVID-19 in a population already engaged with the healthcare system.

## Methods

This prospective cohort study was conducted between 18 May and 16 December 2022 at four (3 PHCs and 1 hospital) government health facilities located in Johannesburg, South Africa, specifically within the COVID-vaccine sites. The study aimed to assess the feasibility and effectiveness of a rapid screening program for diabetes and hypertension. After screening participants for eligibility, obtaining their consent, and enrolling them in the study, various assessments were performed by the study staff. These assessments included a rapid diagnostic test (RDT) for COVID-19, a random glucose test using a glucose meter, blood pressure measurement using a blood pressure cuff, waist circumference measurement using a measuring tape, and height and weight measurements using scales and a height measuring rod. It is important to note that participants were given the choice to opt out of the RDT testing given that this was not a requirement in the government vaccination programme.

Individuals with a first elevated blood pressure measurement had a repeat blood pressure measurement taken after five minutes of rest in a seated position. Individuals who had elevated blood glucose levels, determined by a random glucose level ≥ 11.1 mmol/L or a fasting glucose ≥ 7.0 mmol/L, and/or high blood pressure [12], defined as diastolic blood pressure ≥ 90 mmHg and systolic blood pressure ≥ 140 mmHg [12], were given a written referral that indicated the results of their diabetes and hypertension screening. The study staff strongly encouraged these individuals to seek a confirmation diagnosis of their diabetes and/or hypertension at their local PHC clinic. It is worth noting that a formal laboratory test such as glycated hemoglobin A1c (HbA1c), fasting or random plasma glucose, is required to confirm a diagnosis of diabetes, while repeat blood pressure testing is necessary to confirm a diagnosis of hypertension [12]. Therefore, follow-up evaluations and confirmatory tests were recommended to ensure accurate diagnoses and appropriate management. Participants who received referrals to their local PHC clinic were contacted at regular intervals of 1, 2, and 4 weeks after their enrollment in the study to confirm whether they had visited their PHC clinic for the necessary diagnostic confirmation and further management.

### Study population

All individuals who were literate and 18 years of age or older were invited to participate in the study. Eligible participants were required to possess a mobile phone capable of receiving unstructured supplementary service data, SMS, or WhatsApp messaging. The following categories were excluded from the study: (1) participants who refused consent or were unable to provide informed consent; (2) individuals with contraindications to nasopharyngeal sample collection, which was necessary for the COVID-19 Ag-RDT sample; (3) vulnerable populations as determined by the study personnel, who deemed them inappropriate for inclusion in the study; (4) personnel directly involved in the study’s conduct; (5) participants at significant risk of non-compliance with the protocol provisions, either resulting in harm to themselves or seriously interfering with the validity of the study results.

### Outcomes

Our primary outcomes of interest were as follows:

1. proportion of individuals with active COVID-19 infection among those who received the RDT.
2. proportion of participants with elevated blood glucose levels (i.e., ≥ 11.1 mmol/L if measurement is random; ≥7.0 mmol/L if measurement is equivalent to fasting).
3. proportion of participants with elevated blood pressure indicative of hypertension (i.e., diastolic ≥ 90 mmHg; systolic ≥ 140 mmHg).
4. proportion of participants classified as pre-obese (body mass index (BMI) 25.0-29.9 kg/m^2^), obese (BMI 30.0-39.9 kg/m^2^) and severely obese (BMI ≥ 40 kg/m^2^).
5. proportion of male participants with a waist circumference > 90 cm and female participants > 91.5 cm, indicating risk of metabolic syndrome.
6. proportion of participants with both elevated blood glucose and blood pressure in addition to a waist circumference indicative of metabolic syndrome and/or classified as pre-obese/obese/severely obese.
7. proportion of participants in outcomes 2 and 3 who when contacted stated that they linked to a PHC clinic for diabetes and/or hypertension testing and care.
8. assess predictors of elevated blood glucose, elevated blood pressure and linkage-to-care, separately, using modified Poisson regression.

### Statistical analysis

We employed descriptive statistics to present the clinical and demographic characteristics of the participants in our study. To examine the predictors of elevated blood glucose, elevated blood pressure, and linkage-to-care, we utilized both crude and adjusted modified Poisson regression. Additionally, for our primary outcomes, we stratified the descriptive statistics by biological sex. In the analysis of elevated blood glucose, our model included the following predictors: age (categorized as 18-29.9, 30-39.9, 40-49.9, 50-59.9, and ≥ 60 years), sex, BMI (categorized as < 25 vs. ≥25 kg/m^2^), smoking status (categorized as ever vs. never), elevated blood pressure at enrollment (defined as diastolic ≥ 90 mmHg and systolic ≥ 140 mmHg), previous diabetes diagnosis at enrollment, previous hypertension diagnosis at enrollment, and self-reported HIV status. For the analysis of elevated blood pressure, we adjusted for the same variables mentioned above, except that we replaced elevated blood pressure at enrollment with elevated blood glucose at enrollment as a predictor. For the analysis of linkage-to-care, our model included age (categorized as < 40 vs. ≥40 years due to a smaller sample size), sex, BMI, smoking status, previous diabetes diagnosis at enrollment, previous hypertension diagnosis at enrollment, self-reported HIV status and a variable to identify individuals with one or both conditions at enrollment (categorized as elevated blood glucose only, elevated blood pressure only or both elevated blood glucose and elevated blood pressure). We conducted all statistical analyses using STATA version 16.

## Results

### Cohort

Table 1 provides data on the demographic and health-related characteristics of the study population, which consisted of 1,376 individuals. Among the participants, 638 (46.4%) were male and 738 (53.6%) were female. Most participants were in the 30–39 age group (32.5%), followed by the 18–29 age group (23.0%). The median age for both males and females was 38 years (interquartile range (IQR): 30.0, 47.0). A total of 742 participants (53.9%) were classified as pre-obese, obese, or severely obese. The median BMI for males was 22.7 kg/m^2^ (IQR: 20.5, 26.2 kg/m^2^), indicating a normal weight range, while females had a higher median BMI of 29.2 kg/m^2^ (IQR: 24.6, 34.5 kg/m^2^), suggesting a higher prevalence of overweight or obesity among females. Approximately half of the participants were employed, with a higher percentage of employed females (54.6%) compared to males (44.5%). The majority of participants (71.2%) had received at least one dose of a COVID-19 vaccine prior to enrollment, with a higher vaccination rate among females (74.5%) compared to males (67.4%). The prevalence of ever smoking was 32.5%, with a higher rate observed among males (51.0%) compared to females (16.5%). Among the 240 individuals screened for COVID-19, we did not find a single positive case. The prevalence of HIV was 15.4% overall, and higher among females (20.6%) than males (9.4%) (additional data in Supplementary Table 1), consistent with the prevalence reported in Johannesburg, South Africa. Other comorbid conditions were comparable across sex (mental health conditions (0.4%), cardiovascular disease (1.0%), and asthma (0.8%)).

**Table 1:** Clinical characteristics, demographics and outcomes of participants stratified by sex screened for elevated blood pressure and blood glucose at public sector health facilities in Johannesburg, South Africa (N = 1376)

	Male	Female	Total
	n = 638 (46.4)	n = 738 (53.6)	N = 1376
**Age (years)** (n,%)			
18–29	169 (26.5)	147 (19.9)	316 (23.0)
30–39	185 (29)	249 (33.7)	434 (32.5)
40–49	144 (22.6)	192 (26)	336 (24.4)
50–59	87 (13.6)	104 (14.1)	191 (13.9)
≥ 60	53 (8.3)	46 (6.2)	99 (7.2)
**Age (median; IQR)**	37.0 (29.0, 47.0)	39.0 (31.0, 47.0)	38.0 (30.0, 47.0)
**BMI categories** (n,%)			
underweight (< 18.5 kg/m^2^)	42 (6.6)	18 (2.4)	60 (4.4)
normal (18.5–24.9 kg/m^2^)	392 (61.4)	182 (24.7)	574 (41.7)
pre-obese (25.0-29.9 kg/m^2^)	149 (23.4)	205 (27.8)	354 (25.7)
obese (30.0-39.9 kg/m^2^)	51 (8.0)	255 (34.6)	306 (22.2)
severely obese ( ≥ 40 kg/m^2^)	4 (0.6)	78 (10.6)	82 (6.0)
**Body Mass Index (median; IQR)**	22.7 (20.5, 26.2)	29.2 (24.6, 34.5)	25.7 (21.8, 30.8)
**Employed** (n,%)	284 (44.5)	403 (54.6)	687 (49.9)
**Vaccinated prior to enrollment** (n,%)	430 (67.4)	550 (74.5)	980 (71.2)
**Smoking Status** (n,%)			
never	313 (49.1)	616 (83.5)	929 (67.5)
ever	325 (51.0)	122 (16.5)	447 (32.5)
**Site**			
Yoeville Recreational Centre	87 (13.6)	193 (26.2)	280 (20.4)
Hillbrow Community Health Centre	435 (68.2)	374 (50.7)	809 (58.8)
Clermont Clinic	42 (6.6)	42 (5.7)	84 (6.1)
Charlotte Maxeke Johannesburg Academic Hospital	74 (11.6)	129 (17.5)	203 (14.8)
**Previous diabetes diagnosis at enrollment (self-reported)**	16 (2.5)	28 (3.8)	44 (3.2)
**Previous hypertension diagnosis at enrollment (self-reported)**	55 (8.6)	117 (15.9)	172 (12.5)
**Previous hypertension and diabetes diagnosis at enrollment (self-reported)**	11 (1.8)	17 (2.4)	28 (2.1)
**Other co-morbid conditions at enrollment (self-reported)**			
HIV	60 (9.4)	152 (20.6)	212 (15.4)
Mental health conditions	3 (0.5)	2 (0.3)	5 (0.4)
Cardiovascular disease	6 (0.9)	8 (1.1)	14 (1.0)
Asthma	4 (0.6)	7 (1.0)	11 (0.8)
**Outcomes (n (%))**			
Screened for COVID-19	128 (20.1)	112 (15.2)	240 (17.4)
COVID-19 positivity amongst those screened for COVID-19^4^	0 (0.0)	0 (0.0)	0 (0.0)
Overall elevated blood glucose level^1^	10 (1.6)	12 (1.6)	22 (1.6)
Overall elevated blood pressure indicative of hypertension^2^	75 (11.8)	62 (8.4)	137 (10.0)
Unknown elevated blood glucose level^1,5^	8 (1.3)	4 (0.6)	12 (0.9)
Unknown elevated blood pressure indicative of hypertension^2,6^	61 (10.5)	34 (5.5)	95 (7.9)
Classified as pre-obese, obese, and severely obese	204 (32.0)	538 (72.9)	742 (54.0)
Waist circumference indicative of metabolic syndrome ^3^	189 (29.6)	445 (60.3)	634 (46.1)
≥ 3 or more risk factors above	69 (10.8)	94 (12.7)	163 (11.9)
Linkage-to-care amongst those with elevated blood glucose^7^	4 (50.0)	2 (50)	6 (50)
Linkage-to-care amongst those with elevated blood pressure^8^	36 (61.0)	15 (45.5)	51 (55.4)

^1^≥11.1 mmol/L for random; ≥7.0 mmol/L for fasting

^2^ diastolic ≥ 90 mmHg and systolic ≥ 140 mmHg

^3^ >90 cm (males), > 91.5 cm (females)

^4^ The denominator are those that received a COVID-10 RDT (male n = 128 and female n = 112)

^5^ The denominator are those with no known diabetes at enrollment (male n = 622 and female n = 710)

^6^ The denominator are those with no known hypertension at enrollment (male n = 583 and female n = 621)

^7^ The denominator are those with elevated glucose at enrollment and no known previous diagnosis of diabetes (male n = 8 and female n = 4)

^8^ The denominator are those with elevated blood pressure at enrollment and no known previous diagnosis of hypertension (male n = 61 and female n = 34)

### Primary outcomes

**Fig. 1:**
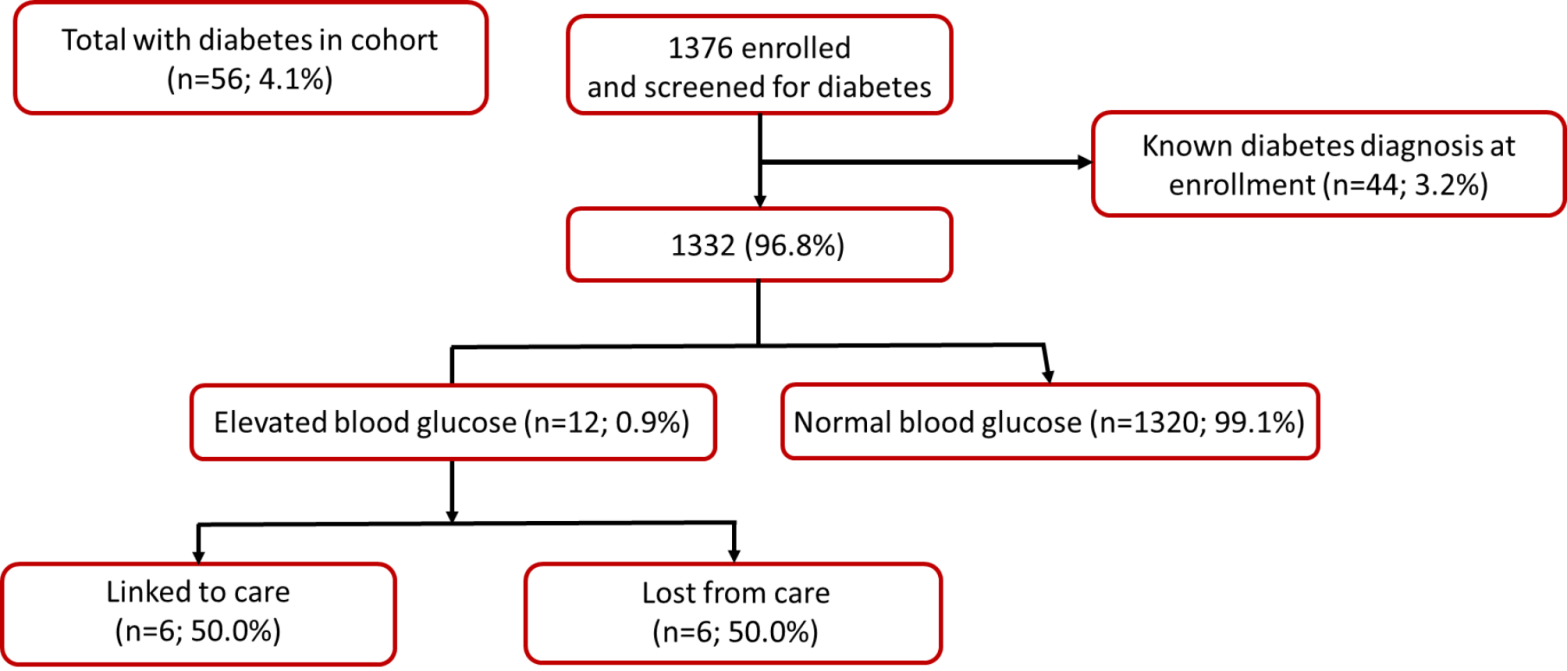
Number and proportion of clients with elevated blood glucose level indicative of diabetes mellitus (DM) (i.e., ≥ 11.1 mmol/L if measurement is random; ≥ 7.0 mmol/L if measurement is equivalent to fasting) amongst those with no known previous diabetes diagnosis at study enrollment (N = 1376)

Combining participants with a previous diabetes diagnosis (n = 44, prevalence: 3.2%, 95% CI: 2.4%, 4.2%) and those with elevated blood glucose at enrollment (n = 12, prevalence: 0.9%, 95% CI: 0.4%, 1.5%), the estimated overall indicative prevalence of diabetes was 4.1% (95% CI: 3.1%, 5.2%) (Table 1; Fig. 1). The 12 participants (or 21% of the total 56 participants) unaware of their elevated blood glucose were predominately male (n = 8; 66%). Women had a slightly higher prevalence of a previous diabetes diagnosis at study enrollment (prevalence: 3.8%, 95% CI: 2.6%, 5.4%) compared to their male counterparts (prevalence: 2.5%, 95% CI: 1.5%, 4.0%). Amongst those with no known diabetes diagnosis, males had slightly higher elevated blood glucose levels at enrollment (1.3%, 95% CI: 0.6, 2.5%) compared to females (0.6%, 95% CI: 0.2, 1.3%). It is worth noting that the proportion of participants with elevated blood glucose at enrollment in our cohort was small. Among the 44 participants with a previous diabetes diagnosis, 10 individuals (22.7%, 95% CI: 12.2%, 36.8%) had elevated blood sugar levels at enrollment, and the majority (n = 8, 80%) were female.

**Fig. 2:**
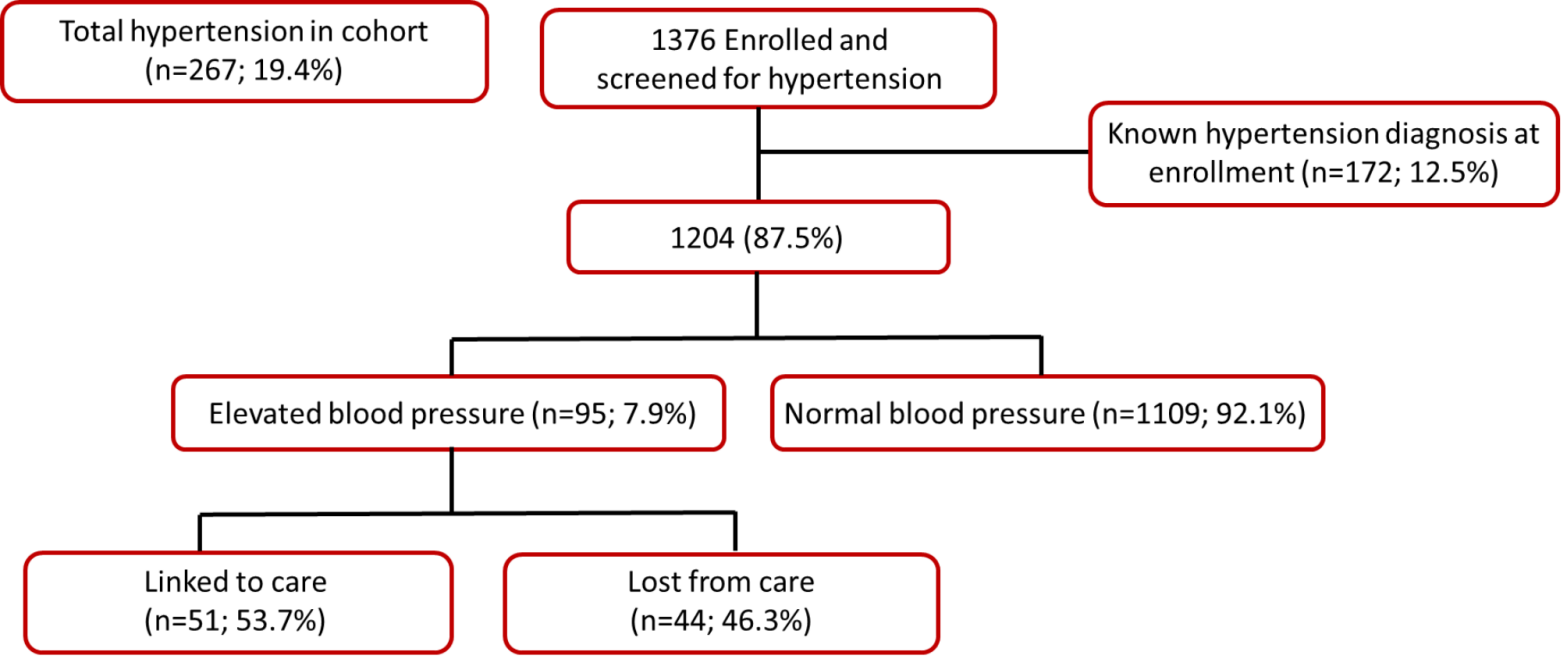
Number and proportion of clients with elevated blood pressure indicative of hypertension (HTN) (i.e. diastolic ≥ 90 mmHg; systolic ≥ 140 mmHg) amongst those with no known previous diagnosis of hypertensive at study enrollment (N = 1376)

When we combine those with known hypertension at study enrollment (n = 172, 12.5%, 95% CI: 10.8, 14.3%) and those with elevated blood pressure (n = 95, 7.9%; 95% CI: 6.5, 9.5%), we get an overall prevalence indicative of hypertension of 19.4% (95% CI: 17.4, 21.6%) (Table 1; Fig. 2). Of the 95 participants (or 35.6% of the total 267 participants) who were unaware of their elevated blood pressure, about two thirds were male (64.2%, 95% CI: 54.2, 73.4%). We found that women (n = 117; 15.9%, 95% CI: 13.4, 18.6%) had a higher prevalence of hypertension diagnosis at study enrollment than their male counterparts (n = 55; 8.6%, 95% CI: 6.6, 11.0%). However, we saw that amongst those with no known hypertension diagnosis, males (10.5%, 95% CI: 8.2, 13.1%) had a higher prevalence of elevated blood pressure compared to females (5.5%, 95% CI: 3.9, 7.5%). A total of 41 (23.8%, 95% CI: 17.9, 30.6%) participants who had a previous diagnosis of hypertension had uncontrolled blood pressure at enrollment, 66.7% (n = 28) of whom were female.

A substantial proportion of our cohort (46.1%, 95% CI: 43.5, 48.7%) consisting of 634 individuals had a waist circumference indicative of metabolic syndrome (> 90 cm for males and > 91.5 cm for females) (Table 1). Notably, the majority of these individuals were female, accounting for 60.3% (n = 445, 95% CI: 66.5, 73.7%). Moreover, 11.9% (95% CI: 10.2, 13.6%) of participants were identified as having 3 or more risk factors - elevated blood glucose and blood pressure along with a high waist circumference and/or fell into the pre-obese/obese/severely obese categories.

Among the participants with elevated blood glucose and no known previous diagnosis of diabetes (n = 12), half of them (n = 6, 50%) successfully linked to care within four weeks of study enrollment, as shown in Table 1; Fig. 1. Similarly, among those with elevated blood pressure and no known hypertension diagnosis (n = 95), more than half (n = 51, 53.6%) were able to link to care. Interestingly, when examining the participants with elevated blood glucose, the proportion of males (n = 4, 50%) seeking PHC services was comparable to females (n = 2, 50.0%). However, among those with elevated blood pressure, a higher percentage of males (n = 36; 61.0%) successfully linked to PHC services compared to females (n = 15; 45.5%), as presented in Table 1.

### Predictors of elevated glucose, elevated blood pressure, metabolic Disease, and linkage-to-care

Table 2 presents the adjusted risk ratio (aRR) estimates with 95% confidence intervals for the outcome of elevated blood glucose. Participants aged ≥ 40 had a higher risk of elevated blood glucose compared to those aged < 40 (aRR: 5.48, 95% CI: 1.18, 25.4). Male participants showed a higher risk of elevated blood glucose, although the association was imprecise (aRR: 2.36, 95% CI: 0.92, 6.04), compared to females. Participants with a BMI ≥ 25.0 kg/m^2^ (vs. <25.0 kg/m^2^) (aRR: 11.8, 95% CI: 1.24, 112.8) and those with a previous diagnosis of diabetes (aRR: 14.5, 95% CI: 5.27, 39.9) had a higher risk of elevated blood glucose. The other covariates were not predictors of elevated blood glucose at study enrollment.

**Table 2:** Crude and adjusted risk ratios assessing predictors of elevated blood glucose and blood pressure (n = 1376)

	elevated blood glucose		elevated blood pressure	
	RR (95% CI)	aRR (95% CI)	RR (95% CI).	aRR (95% CI)
**Age (years)**				
< 40	**ref**	**ref**	**ref**	**ref**
≥ 40	13.6 (3.17, 58.0)	5.48 (1.18, 25.4)	2.60 (1.83, 3.70)	1.76 (1.18, 2.63)
**Sex**				
Female	**Ref**	**ref**	**ref**	**Ref**
Male	0.96 (0.42, 2.23)	2.36 (0.92, 6.04)	1.40 (1.00, 1.96)	1.80 (1.23, 2.64)
**Previous hypertension diagnosis**				
No	**ref**	**ref**	**ref**	**ref**
Yes	4.00 (1.68, 9.53)	0.72 (0.27, 1.91)	3.09 (2.15, 4.45)	2.28 (1.51, 3.45)
**Previous diabetes diagnosis**				
No	**ref**	**ref**	**ref**	**ref**
Yes	25.2 (10.9, 58.4)	14.5 (5.27, 39.9)	1.39 (0.61, 31.4)	0.53 (0.22, 1.29)
**Elevated blood pressure at enrollment**				
No	**ref**	**ref**	--	--
Yes	2.67 (0.98, 7.21)	1.77 (0.63, 4.94)	--	--
**Elevated blood glucose diagnosis at enrollment**				
No	--	--	**ref**	**ref**
Yes	--	--	2.33 (0.95, 5.69)	1.52 (0.59, 3.89)
**Body Mass Index**				
< 25.0 kg/m^2^	**ref**	**ref**	**ref**	**ref**
≥ 25.0 kg/m^2^	17.9 (2.41, 133.0)	11.8 (1.24, 112.8)	1.64 (1.15, 2.33)	0.19 (0.66, 1.79)
**Waist circumference**				
Low risk	**ref**	**ref**	**ref**	**ref**
High risk	7.4 (2.2, 25.0)	1.15 (0.29, 4.63)	2.17 (1.53, 3.08)	1.82 (1.11, 2.98)
**HIV**				
No	**ref**	**ref**	**ref**	**ref**
Yes	0.82 (0.24, 2.76)	0.96 (0.27, 3.39)	0.88 (0.55, 1.42)	0.88 (0.54, 1.44)
**Smoking Status**				
Never	**ref**	**ref**	**ref**	**ref**
Ever (current or former)	0.61 (0.23, 1.66)	0.62 (0.21, 1.89)	0.95 (0.66, 1.36)	1.05 (0.71, 1.56)

BMI, body mass index

For the outcome of elevated blood pressure, we found participants aged ≥ 40 had a higher risk of elevated blood pressure compared to those aged < 40 (aRR: 1.76, 95% CI: 1.18, 2.63) (Table 3). Male participants also showed a higher risk of elevated blood pressure compared to females (aRR: 1.80, 95% CI: 1.23, 2.64). Having a previous diagnosis of hypertension (aRR: 2.28, 95% CI: 1.51, 3.45) and having a high waist circumference (aRR: 1.82, 95% CI: 1.11, 2.98) were associated with a higher risk of elevated blood pressure. The other covariates were not associated with elevated blood pressure at enrollment.

For the outcome of linkage-to-care (Table 3), although imprecise, having both elevated blood glucose and elevated blood pressure at enrollment compared to just having one of those conditions alone increased the probability of linkage to follow-up care (aRR 1.28; 95% CI: 0.57, 2.84). The other covariates were not associated with linkage-to-care.

**Table 3:** Predictors of linkage-to-care amongst those with elevated blood glucose and/or blood pressure (n = 164)

	Linked to Care	
	RR (95% CI)	aRR (95% CI)
**Age**		
< 40	**ref**	**ref**
≥ 40	0.91 (0.70, 1.19)	0.91 (0.65, 1.28)
**Comorbidities**		
Elevated blood pressure	**ref**	**ref**
Elevated blood glucose	0.88 (0.55, 1.41)	0.95 (0.55, 1.64)
Both	1.24 (0.58, 2.64)	1.28 (0.57, 2.84)
**Previous hypertension diagnosis**		
No	**ref**	**ref**
Yes	0.88 (0.64, 1.20)	0.96 (0.64, 1.43)
**Previous diabetes diagnosis**		
No	**ref**	**ref**
Yes	0.82 (0.50, 1.32)	0.87 (0.46, 1.67)
**Sex**		
Female	**ref**	**ref**
Male	0.95 (0.74, 1.23)	0.95 (0.67, 1.35)
**BMI**		
< 25.0 kg/m^2^	**ref**	**ref**
≥ 25.0 kg/m^2^	1.02 (0.78, 1.34)	1.07 (0.73, 1.67)
**HIV**		
No	**ref**	**ref**
Yes	1.02 (0.69, 1.52)	1.06 (0.67, 1.70)
**Smoking Status**		
Never	**ref**	**ref**
Ever (current or former)	1.07 (0.82, 1.39)	1.08 (0.76, 1.54)

## Discussion

The primary objective of our research was to evaluate the feasibility and effectiveness of simultaneous screening for diabetes, hypertension, and COVID-19 during a government-led COVID-19 vaccination drive. Early intervention and management for these conditions are cost-effective; however, their widespread application has been hindered by an overwhelmed healthcare system, a situation intensified by the COVID-19 pandemic. By leveraging existing COVID-19 screening infrastructures at government vaccination centers in Johannesburg, South Africa, we demonstrated the viability of conducting preliminary checks for elevated blood pressure and blood glucose levels outside conventional healthcare venues. In a context where individuals were already seeking health measures, we provided them with easy-to-access health insights for conditions that are promptly addressable. This approach holds notable importance for the population, except for people living with HIV who might already be frequenting PHC centers for HIV management. However, ensuring a swift transition to suitable healthcare services remains a challenge. Even as the role of community-based COVID-19 vaccinations have started to diminish, our screening methodology stands poised for smooth integration into existing or upcoming health initiatives steered by medical professionals.

During our eight-month study, we screened 1,376 individuals, uncovering an overall diabetes prevalence of 4.1%. This rate combines those with a prior diabetes diagnosis (3.2%) and those detected with elevated blood glucose upon enrollment (0.9%). Remarkably, this rate falls significantly below previously documented figures for South Africa (11%) [2, 3], sub-Saharan Africa (7.2%) [10], and even our prior investigation in Johannesburg taxi ranks, which registered a 7.1% prevalence [11] utilizing the same screening method. One potential explanation for this disparity might be the proactive health-seeking behavior of our cohort, especially their pursuit of COVID-19 vaccinations at medical establishments. In our study’s initial phase, both females (3.8%) and males (2.5%) exhibited comparable rates of previously diagnosed diabetes. Furthermore, when setting aside those already diagnosed, our findings showed similar proportions of elevated blood glucose levels between the genders, consistent with previous studies [13]. It is noteworthy that 22.7% of participants with a prior diabetes diagnosis had elevated blood glucose during enrollment, with females constituting 80% of this subset. This aligns with South Africa’s historically documented poor glycaemic control in adults living with diabetes, which has ranged between 7.6 and 83.6% [14–16], and currently stands at 77.7% [17].

Regarding hypertension, our study estimated an indicative prevalence of 19.4% (combining previous hypertension diagnosis of 12.5% with those with an elevated blood pressure at enrollment 6.9%), which is comparable to those previously reported out of South Africa [18], however, lower than what was reported in our previous taxi rank study using the same tool (27.9%) [11]. The prevalence of previous hypertension diagnosis at study enrollment was higher in females (15.9%) than males (8.6%). However, among individuals with no known previous hypertension diagnosis, males (10.5%) had a higher prevalence of elevated blood pressure compared to females (5.5%), also consistent with previous work [11, 18]. These differences by sex in the prevalence of hypertension could be associated with higher magnitudes of obesity and physical inactivity among women in our study population [18]. Additionally, 23.8% of participants who had a previous diagnosis of hypertension had uncontrolled blood pressure at enrollment, 66.7% of whom were female. Our estimates are in line with those previously reported out of South Africa (13.5 − 75.5% [19, 20], whilst figures ranging between 19.0 and 56.0% have been reported for hypertension control [21].

Metabolic syndrome, encompassing multiple cardiovascular risk factors, was once considered uncommon in Africa. However, recent evidence indicates an escalating prevalence, especially among older age groups, which is predominantly attributed to lifestyle changes such as decreased physical activity and the replacement of a fruit-rich diet with more calorie-dense foods [22]. In a 2017 study conducted in South Africa, the estimated prevalence of metabolic syndrome was 46.3% in females and 29.3% in males [23]. Notably, a significant proportion of our study participants (46.1%) exhibited a large waist circumference, which is strongly associated with metabolic syndrome. This was more prevalent among females (60.3%) compared to males (29.6%), consistent with previous research in South Africa [24]. Importantly, our findings diverged significantly from our previous taxi rank study, where the overall prevalence of metabolic syndrome was much lower (0.7%), including 0.8% for females and 0.6% for males [11]. Furthermore, 11.9% of participants (12.7% females, 10.8% males) in our cohort displayed three or more indicators of metabolic syndrome, including elevated blood glucose and blood pressure, along with a high waist circumference or falling into pre-obese, obese, or severely obese categories.

One of the most intriguing discoveries in our study was the identification of an additional 12 individuals (21.4% of the total 56 participants potentially living with diabetes) and an additional 95 individuals (35.6% of the total 267 participants potentially living with hypertension) who were potentially newly diagnosed with either condition. This finding means that 7.8% of the screened participants were previously unaware of their elevated blood glucose or blood pressure levels. Comparing this result to our previous research in the taxi rank study [11], we observed a lower percentage of participants unaware of their conditions. This discrepancy may be attributed to the differences in the populations accessed during the two studies. Notably, while the cohort included in our taxi rank study was itinerant, passing through the ranks on their way to or from work or other engagements, the current cohort was recruited at healthcare facilities and thus consisted of individuals already accessing the healthcare system. This potentially led to a higher likelihood of being screened and treated for diabetes and hypertension. Similar patterns have been observed in other studies focused on diabetes [17, 25] and hypertension [26, 27] in sub-Saharan Africa. It is worth noting that more than 50% of people living with diabetes in sub-Saharan Africa remain unaware of their condition [4, 17, 28], while 7–56% of people living with hypertension are unaware of their blood pressure status [18, 29]. Further analysis of our data revealed that those who were unaware of their blood glucose or blood pressure levels were predominantly male, consistent with what was found in our taxi rank study [11]. This observation might be a result of men being less engaged in the healthcare system compared to women [17], potentially leading to lower screening rates for these conditions.

One of the primary objectives of our study was to evaluate the linkage to care for individuals diagnosed with diabetes and hypertension. Among participants with elevated blood glucose and/or blood pressure, approximately 50% successfully connected with primary healthcare services within four weeks of study enrollment. The linkage to care showed the same rate among males (50%) and females (50%) with elevated blood glucose. However, a higher proportion of males (61.0%) with elevated blood pressure sought medical care compared to females (45.5%). Remarkably, these linkage rates are higher than those previously reported in the taxi rank study (elevated blood glucose linkage 30.0%; elevated blood pressure linkage 16.3%) [11]. This disparity is likely attributable to the participants in our current study were already utilizing the healthcare system for COVID-19 vaccination, providing an optimal opportunity for opportunistic screening and early detection of NCDs. The findings from our study strongly suggest that conducting opportunistic screening at COVID-19 vaccination sites can effectively identify individuals at risk of NCDs and facilitate their access to necessary follow-up care. However, it is essential to focus on improving the linkage to care, particularly for individuals with elevated blood pressure, to ensure proper management and control of hypertension.

Our study has several notable strengths. Firstly, we demonstrate that any interaction with the healthcare system, no matter how incidental, can be utilized as an opportunity to screen for additional diseases or co-morbidities. This finding is significant as it highlights the potential for integrating screening efforts into routine healthcare encounters and other large-scale health programmes. Furthermore, our results may have broader applicability to the general population in Southern Africa and potentially extend to other regions in sub-Saharan Africa with high obesity prevalence. However, it is important to acknowledge the limitations of our study. Firstly, it should be noted that a single measurement of blood glucose or blood pressure is insufficient to diagnose diabetes or hypertension. Definitive diagnoses require further laboratory tests and clinical evaluations, as well as linkage to the healthcare system for comprehensive diagnosis and treatment. Secondly, the prior diagnoses of diabetes and hypertension in our study relied on self-reporting by participants, which introduces the possibility of response bias. Lastly, there may be a recruitment bias due to our selection of participants engaging in the health care system for vaccination, these patients may have better health seeking behavior than the general population.

## Conclusion

In summary, our study shows that it is practical and effective to screen for diabetes and hypertension at government COVID-19 vaccination sites in Johannesburg, South Africa. By using these existing health touchpoints, we were able to find individuals at risk and connect them to care more effectively than in our previous study. Our results suggest that it is possible to include such screenings in everyday healthcare routines, making it a practical approach for future health screenings. When we look at our previous taxi rank study [11], it seems that broadening such initiatives to high-footfall community locales might help identify more people with diabetes and hypertension. However, these individuals might be less inclined to seek further care. There is a clear need to improve how we connect people to care to ensure they get the ongoing support they need. Despite its limitations, our study offers useful insights for addressing gaps in care for chronic diseases in places with limited resources. Early detection and follow-up care are essential to manage these health issues effectively.

### Electronic supplementary material

Below is the link to the electronic supplementary material.

Supplementary Material 1

## Data Availability

Anonymized datasets used and/or analysed during the current study are available at OpenBU (https://open.bu.edu/), Boston University’s open access repository.
