# Supplementary material for: Integration of point-of-care screening for type 2 diabetes mellitus and hypertension into the COVID-19 vaccine programme in Johannesburg, South Africa
Source: BMC Public Health. 2023 Nov 20;23:2291. doi: 10.1186/s12889-023-17190-6 (PMC10662646; doi:10.1186/s12889-023-17190-6)
Supplement: Supplementary file 1 — Supplementary Material 1 [file 12889_2023_17190_MOESM1_ESM.pdf]

Supplementary Table 1. Clinical characteristics, demographics and outcomes of participants stratified by HIV status screened elevated blood pressure and blood glucose at public sector health facilities in Johannesburg, South Africa (N=1376)

	PLWOH n=1153 (84.6)	PLWH n=223 (15.4)	Total N=1376
**Age (years)** (n,%)			
18-29	307 (26.6)	9 (4.0)	316 (23.0)
30-39	366 (31.7)	68 (30.5)	434 (31.5)
40-49	243 (21.1)	93 (41.7)	336 (31.5)
50-59	148 (12.8)	43 (19.3)	191 (13.9)
$\geq$60	89 (7.7)	10 (4.5)	99 (7.2)
**Age (median; IQR)**	37.0 (29.0, 47.0)	43.0 (37.0, 49.0)	38.0 (30.0, 47.0)
**BMI categories** (n,%)			
underweight (<18.5 kg/m^2)	55 (4.8)	7 (3.1)	62 (4.5)
normal (18.5-24.9 kg/m^2)	484 (42.0)	88 (39.5)	572 (41.6)
pre-obese (25.0-29.9 kg/m^2)	294 (25.5)	60 (26.9)	354 (25.7)
obese (30.0-39.9 kg/m^2)	253 (21.9)	53 (23.8)	306 (22.2)
severely obese (>=40 kg/m^2)	67 (5.8)	15 (6.7)	82 (6.0)
**Body Mass Index (median; IQR)**	25.5 (21.7, 30.6)	26.4 (22.5, 31.5)	25.7 (21.8, 30.8)
**Employed** (n,%)	588 (51.0)	99 (44.4)	687 (50.0)
**Vaccinated prior to enrollment** (n,%)	816 (70.8)	164 (73.5)	980 (71.2)
**Smoking Status** (n,%)			
never	779 (67.6)	150 (67.3)	929 (67.5)
Ever	374 (32.4)	73 (32.7)	447 (32.5)
**Site**			
Yoeville Recreational Centre	221 (19.2)	59 (26.5)	280 (20.4)
Hillbrow Community Health Centre	667 (57.9)	142 (63.7)	809 (58.8)
Clermont Clinic	76 (6.6)	8 (3.6)	84 (6.1)
Charlotte Maxeke Johannesburg Academic Hospital	189 (16.4)	14 (6.3)	203 (14.8)
**Previous diabetes diagnosis at enrollment (self-reported)**	40 (3.5)	4 (1.8)	44 (3.2)
**Previous hypertension diagnosis at enrollment (self-reported)**	143 (12.4)	29 (13.0)	172 (12.5)
**other co-morbid conditions at enrollment (self-reported)**			
mental health	4 (0.4)	1 (0.5)	5 (0.4)
cardiovascular disease	12 (1.0)	2 (0.9)	14 (1.0)
asthma	11 (1.0)	0 (0.0)	11 (0.8)

Outcomes (n (%))			
screened for COVID-19	204 (17.7)	36 (16.1)	240 (17.4)
COVID-19 positivity amongst screened for COVID-19[4]	0 (0.0)	0 (0.0)	0 (0.0)
overall elevated blood glucose level[1]	19 (1.7)	3 (1.4)	22 (1.6)
overall elevated blood pressure indicative of hypertension[2]	117 (10.2)	20 (9.0)	137 (10.0)
unknown elevated blood glucose level[1,5]	11 (1.0)	1 (7.7)	12 (0.9)
unknown elevated blood pressure indicative of hypertension[2,6]	84 (7.3)	11 (4.9)	95 (6.9)
classified as pre-obese, obese, and severely obese	614 (53.3)	128 (57.4)	742 (53.9)
waist circumference indicative of metabolic syndrome [3]	518 (44.9)	116 (52.0)	634 (46.1)
≥3 or more risk factors above	143 (12.4)	20 (9.0)	163 (11.9)
Linkage-to-care amongst those with elevated blood glucose[7]	5 (45.5)	1 (100)	6 (50.0)
Linkage-to-care amongst those with elevated blood pressure[8]	45 (54.9)	6 (60.0)	51 (55.4)

[1] ≥11.1 mmol/L for random; ≥7.0 mmol/L for fasting

[2] diastolic ≥90 mmHg and systolic ≥140 mmHg

[3] >90 cm (males), >91.5 cm (females)

[4] denominator is among those that received a COVID-10 RDT (male n=204 and female n=36)

[5] denominator is among those with no known diabetes at enrollment (male n=1113 and female n=219)

[6] denominator is among those with no known hypertension at enrollment (male n=1010 and female n=194)

[7] denominator is among those with elevated glucose at enrollment and no known previous diagnosis of diabetes (male n=11 and female n=1)

[8] denominator is among those with elevated blood pressure at enrollment and no known previous diagnosis of hypertension (male n=84 and female n=11)
